# Peripheral artery disease, abnormal ankle-brachial index, and prognosis in patients with acute coronary syndrome

**DOI:** 10.3389/fcvm.2022.902615

**Published:** 2022-09-06

**Authors:** Anat Berkovitch, Zaza Iakobishvili, Shmulik Fuchs, Shaul Atar, Omri Braver, Alon Eisen, Michael Glikson, Roy Beigel, Shlomi Matetzky

**Affiliations:** ^1^Division of Cardiology, Leviev Heart and Vascular Center, Chaim Sheba Medical Center, Tel Hashomer, Israel; ^2^Sackler School of Medicine, Tel Aviv University, Tel Aviv, Israel; ^3^Rabin Medical Center, Petah Tikva, Israel; ^4^Department of Cardiology, Yitzhak Shamir Medical Center, Tel Aviv, Israel; ^5^Department of Cardiology, Galilee Medical Center, Nahariya, Israel; ^6^Azrieli Faculty of Medicine, Bar-Ilan University, Safed, Israel; ^7^Department of Cardiology, Soroka University Medical Center and Faculty of Health Sciences, Ben-Gurion University of the Negev, Beer Sheva, Israel; ^8^Integrated Heart Center, Shaare Zedek Medical Center, Jerusalem, Israel

**Keywords:** acute coronary syndrome, peripheral vascular disease, peripheral arterial disease (PAD), vascular disease (PVD), ankle brachial blood pressure index, claudication

## Abstract

**Objectives:**

Ankle-brachial index (ABI) is an independent prognostic marker of cardiovascular events among patients with coronary artery disease (CAD). We aimed to investigate the outcome of patients hospitalized with acute coronary syndrome (ACS) and abnormal ABI.

**Approach and results:**

ABI was prospectively measured in 1,047 patients hospitalized due to ACS, who were stratified into three groups, namely, those with clinical peripheral artery disease (PAD) (*N* = 132), those without clinical PAD but with abnormal (< 0.9) ABI (subclinical PAD; *N* = 148), and those without clinical PAD with normal ABI (no PAD; *N* = 767). Patients were prospectively followed for 30-day major adverse cardiovascular event (MACE) and 1-year all-cause mortality. The mean age was 64 years. There was a significant gradual increase throughout the three groups in age, i.e., the incidence of prior stroke, diabetes mellitus, and hypertension (p for trend = 0.001 for all). The in-hospital course showed a gradual rise in the incidence of complications with an increase in heart failure [2.5, 6.1, and 9.2%, (p for trend = 0.001)] and acute kidney injury [2, 4.1, and 11.5%, (p for trend = 0.001)]. At day 30, there was a stepwise increase in MACE, such that patients without PAD had the lowest rate, followed by subclinical and clinical PADs (3.5, 6.8, and 8.1%, respectively, p for trend = 0.009). Similarly, there was a significant increase in 1-year mortality from 3.4% in patients without PAD, through 6.8% in those with subclinical PAD, to 15.2% in those with clinical PAD (p for trend = 0.001).

**Conclusion:**

Subclinical PAD is associated with poor outcomes in patients with ACS, suggesting that routine ABI screening could carry important prognostic significance in these patients regardless of PAD symptoms.

## Introduction

In patients with stable coronary artery disease (CAD), concomitant peripheral arterial disease (PAD) independently predicts worse short- and long-term clinical outcomes, including higher mortality (1). Yet, many patients with PAD are asymptomatic and, therefore, under-diagnosed (2). While the prevalence of symptomatic PAD in patients with CAD is about 10%, it is estimated that asymptomatic PAD affects at least two times as many patients with CAD (3–5).

Ankle-brachial index (ABI) represents the ratio between the systolic blood pressure measured at the ankle and the brachial arteries (6, 7). While ABI is used to evaluate the presence and severity of PAD in patients with symptoms of claudication or non-healing lower-extremity wounds, it is also able to detect a significant PAD in asymptomatic patients (8). Accordingly, prior studies have shown that ABI is an independent prognostic marker for cardiovascular events among patients with stable CAD (9–13). Furthermore, it was found that early recognition and treatment of PAD can help to reduce morbidity and mortality and prevent cardiovascular events (14, 15).

In contrast, only limited data exist regarding the significance of PAD detection in patients with ACS, and even less regarding ABI in ACS patients with asymptomatic PAD (1, 16–21), most of which are non-conclusive. We aimed to prospectively evaluate the prevalence and clinical significance of pathological ABI in a large group of consecutive patients hospitalized with ACS without symptomatic or known PAD.

## Materials and methods

### Study population

We prospectively investigated 1,047 consecutive patients suffering from an ACS event. The patients were drawn from the Acute Coronary Syndrome Israeli Survey (ACSIS), which has been previously described (22). In short, the ACSIS is a national survey that was conducted over a 2-month period every other year from 2000 to 2018. Data were collected prospectively from all patients discharged with the diagnosis of acute myocardial infarction in 22 coronary care units and cardiology wards operating in Israel. In 2018, ABI was added to the survey and performed on all patients enrolled. In addition, only patients hospitalized in 2018 were included in this study. The recorded discharge diagnoses were determined by the attending physicians based on clinical, electrocardiographic, and biochemical criteria. As part of their evaluation, all patients underwent an ABI test.

### Inclusion and exclusion criteria

The study comprised 1,047 consecutive patients hospitalized due to an AMI event. The diagnosis of clinical PAD was prospectively determined upon admission based on self-reporting symptoms compatible with intermittent claudication, prior imaging demonstrating peripheral occlusive disease, peripheral revascularization, or prior amputation due to vascular occlusive disease.

In each of the patients, the systolic blood pressure from the right and left brachial arteries, the right and left posterior tibial arteries, and the dorsalis pedis arteries was measured in order to calculate the ABI. The ratio between the systolic blood pressure at the lower extremities and the higher systolic pressure in the right or left arm was regarded as the ABI value. A value of < 0.9 on at least one side was considered to be an abnormal ABI.

Patients were categorized into three groups, namely, patients with clinical PAD (*N* = 132), patients without clinical PAD but with abnormal ABI (subclinical PAD; *N* = 148), and patients without clinical PAD with normal ABI (no PAD; *N* = 767) (Supplementary Figure 1).

Baseline information including risk factors for atherosclerotic CAD, medical cardiovascular history, and baseline medications were prospectively recorded on pre-specified forms. A history of ischemic heart disease, heart failure, a cerebrovascular attack, hypertension, diabetes mellitus, dyslipidemia, and smoking status were all based on known diagnoses and/or concurrent diabetic or lipid-lowering medications. The renal function was evaluated using the Cockcroft–Gault equation.

The regional ethical review board at each site approved the trial protocol, and the trial was conducted according to the principles of the Declaration of Helsinki. Institutional review board approval was obtained from all the participating centers and all patients provided signed informed consent to participate in the study (Helsinki number for the correct study 4486-17).

In-hospital and 30-day outcomes were prospectively recorded and ascertained by hospital chart review, telephone contact, and clinical follow-up data. Mortality data during hospitalization and at 30-day and 1-year post-hospitalization were obtained from patients’ hospital charts and the Ministry of Health register.

The primary outcome of this study was 1-year all-cause mortality. The secondary outcome was 30-day major adverse cardiovascular events (MACEs). A 30-day MACE was defined as recurrent myocardial infarction, stent thrombosis, unstable angina requiring urgent revascularization, stroke, and/or cardiovascular mortality. Secondary analyses included in-hospital complications and outcomes at 30 days for each of the individual MACE components.

### Statistical analysis

The three study groups were tested with a chi-square test for trend for categorical variables, with the analysis of variance with 1 degree of freedom for comparison of normally distributed continuous variables, and with Kendall rank correlation for non-normal distribution. For the comparison of two groups, chi-square, *t*-test, or Mann–Whitney–Wilcoxon test were used as appropriate for categorical variables, normal, or non-normal distributed continuous variables.

The probability of 1-year mortality and 30-day MACE according to the three pre-specified PAD groups was estimated and graphically displayed.

To explore the relationship between survival, the study groups, and other explanatory covariates, both univariable and multivariable Cox proportional hazard models for 1-year mortality were performed. For the purpose of the analysis, clinical PAD and subclinical PAD were merged into one group and were compared to the non-PAD group. The multivariable analysis was adjusted for age, gender, and any cardiovascular risk factors that found to be statistically significant (p for trend < 0.05) in the baseline univariable analysis.

To obtain a better cutoff point for the ABI, a receiver operating characteristic (ROC) curve was plotted, and a threshold was calculated according to Youden’s J statistic for max (sensitivity and specificity). To further explore the relationship between survival and the different ABI cutoff points, univariable Cox proportional hazard models were performed, each model for each of the cutoff points (1, 0.9, 0.8, and 0.7).

A statistical significance was accepted for a two-sided *p* < 0.05. The statistical analyses were performed using R statistics version 3.5.3.

## Results

The study population comprised 1,047 consecutive patients with AMI, of whom 20% were women, with a median age of 64 years [interquartile range (IQR) 55, 72]. Of the entire patient cohort, 132 (13%) patients had clinical PAD, 148 (14%) patients had subclinical PAD (ABI < 0.9), and 767 (73%) patients had no evidence of PAD. Noteworthy, among the 280 patients with either clinical or non-clinical PAD, only less than half [132 (47%)] had symptoms and/or a history suggestive of PAD. Interestingly, patients with symptomatic PAD had a significantly higher ABI compared to those with subclinical PAD [0.93 (0.67–0.8) vs. 0.78 (0.72–0.85), *p* < 0.001].

Throughout the three study groups, there was a gradual and significant rise in patients’ age, cardiovascular risk factors including diabetes mellitus, hypertension, dyslipidemia, and smoking history, as well as incidences of chronic renal impairment from patients without PAD to those with subclinical and clinical PADs (p for trend = 0.001 for all). Similarly, there was a significant gradation in the incidence of prior stroke, as well as prior coronary artery intervention, prior ACS, and baseline ischemic heart disease (Table 1).

**TABLE 1:** Population baseline characteristics.

	No PAD *N* = 767	Subclinical PAD *N* = 148	Clinical PAD *N* = 132	*P* for trend
Age – mean (SD) years	62.68 (12.36)	65.03 (13.14)	70.83 (10.49)	<0.001
Males – N (%)	632 (82.4)	118 (79.7)	108 (81.8)	0.677
Body mass index – mean (SD)	28.21 (4.57)	27.89 (4.52)	27.27 (5.14)	0.034
Past smoker – N (%)	124 (16.2)	29 (19.6)	41 (31.1)	<0.001
Current smoker – N (%)	340 (44.3)	70 (47.3)	56 (42.4)	0.903
Family history of CAD – N (%)	242 (36.0)	39 (30.2)	27 (27.3)	0.048
Dyslipidemia – N (%)	520 (68.0)	106 (72.1)	121 (91.7)	<0.001
Hypertension – N (%)	479 (62.6)	110 (74.8)	119 (90.2)	<0.001
Diabetes mellitus – N (%)	284 (37.1)	69 (46.6)	84 (63.6)	<0.001
HbA1c (median [IQR])	5.90 [5.44, 6.60]	5.90 [5.40, 7.10]	6.70 [5.89, 8.20]	<0.001
Chronic renal failure – N (%)	52 (6.8)	25 (16.9)	50 (37.9)	<0.001
Prior ACS events	249 (32.5)	64 (43.2)	86 (65.2)	<0.001
Ischemic heart disease	271 (35.4)	66 (44.6)	94 (71.2)	<0.001
Heart failure – N (%)	63 (8.2)	13 (8.8)	39 (29.5)	<0.001
Prior revascularization – N (%)	245 (32.0)	57 (38.5)	84 (64.6)	<0.001
Prior stroke/TIA – N (%)	47 (6.1)	18 (12.2)	34 (26.0)	<0.001
Aspirin	89 (75.4)	64 (48.9)	290 (47.8)	<0.001
P2Y12 inhibitors	43 (41.7)	30 (32.6)	85 (81.7)	<0.001
Warfarin	1 (1.3)	3 (3.9)	10 (2.5)	0.718
Apixaban	12 (14.3)	3 (3.9)	13 (3.2)	<0.001
Dabigatran	0 (0)	0 (0)	4 (1.0)	0.242
Rivaroxaban	5 (6.2)	2 (2.7)	4 (1.0)	0.002
C-reactive protein (mg/dl)	12.16 [2.73, 81.30]	5.30 [2.18, 16.20]	5.44 [1.66, 15.50]	0.004

CAD, coronary artery disease; IQR, inter-quartile range; PAD, peripheral artery disease; ACS, acute coronary syndrome; TIA, transient ischemic attack; SD, standard deviation.

### Clinical and angiographic findings

The in-hospital findings according to the pre-specified study groups are summarized in Table 2. ST-elevation myocardial infarction was more prevalent in patients without PAD, followed by patients with subclinical PAD (p for interaction < 0.001). No significant differences were recorded in peak Troponin I level [40 ng/L vs. 28 ng/L vs. 224 ng/L (p for trend 0.4)].

**TABLE 2:** Clinical characteristics.

	Clinical PAD *N* = 132	Subclinical PAD *N* = 148	No PAD *N* = 767	*P* for trend
STEMI (discharge diagnosis) – N (%)	27 (20.5)	53 (35.8)	346 (45.1)	<0.001
Admin SBP (mmHg) mean (SD)	143 (28)	148 (28)	144 (26)	0.86
Admin DBP (mmHg) mean (SD)	77 (15)	83 (16)	83 (15)	0.007
Admin Heart rate (bpm) [mean (SD)]	81.76 (22.37)	80.58 (18.47)	79.05 (17.40)	0.364
**Ejection Fraction category**				
Normal (> = 50%) – N (%)	39 (33.6)	63 (49.2)	339 (51.1)	0.001
Mild (40–49%) – N (%)	33 (28.4)	35 (27.3)	213 (32.1)	0.288
Moderate (30–39%) N (%)	27 (23.3)	23 (18.0)	92 (13.9)	0.007
Severe (< 30%) – N (%)	17 (14.7)	7 (5.5)	20 (3.0)	<0.001
Killip class of ≥ 2- N (%)	28 (23.1)	14 (10.7)	44 (6.2)	<0.001

DBP, diastolic blood pressure; PAD, peripheral artery disease; SBP, systolic blood pressure; STEMI, ST-elevation myocardial infarction.

Compared to patients with symptomatic PAD (86%), those without symptomatic PAD were more likely to have undergone coronary angiography (*p* = 0.034) irrespective of whether they had pathological (94%) or normal ABI (96%).

The extent of CAD as reflected by the incidence of multivessel CAD increased gradually from patients without PAD to those with subclinical and symptomatic PAD (43, 57, and 64%; p for trend < 0.01). Noteworthy, among patients without clinical PAD, pathological ABI was associated with a higher incidence of multi-vessel disease (57 vs. 43%, *p* = 0.004).

While the rate of referrals to coronary artery bypass graft (CABG) within the first 30 days following the index event was similar (9.9% both in patients with clinical and subclinical PADs and 5.1% in patients without PAD, p for trend = 0.06), there were a significantly higher number of patients who underwent PCI from those with clinical PAD (62.5%) and subclinical PAD (64%) as compared to those without PAD (77%, p for trend = 0.002).

### In-hospital course

**FIGURE 1:**
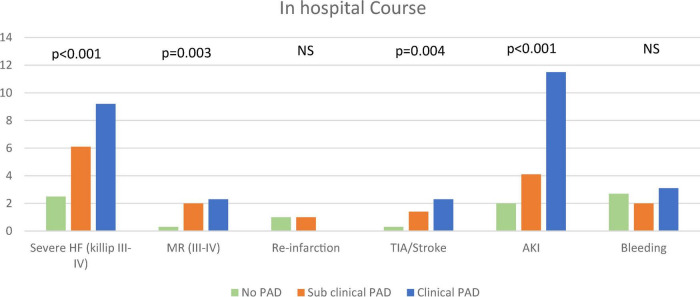
In-hospital course. The figure demonstrates the frequency of in-hospital complications according to the pre-specified groups. The chi-square test for trend was used. AKI, acute kidney injury; MR, mitral regurgitation; PAD, peripheral arterial disease; TIA, transient ischemic attack.

As shown in Figure 1, the three study groups also showed a gradual increase in the incidence of in-hospital complications, including severe heart failure (Killip II-IV: 2.5, 6.1, and 9.2%, p for trend = 0.001), acute kidney injury (2, 4.1, and 11.5%, p for trend = 0.001), and stroke and/or transient ischemic attack (TIA) (0.3, 1.4, and 2.3%, p for trend = 0.004).

Interestingly, there were no differences in the incidence of major and/or clinically relevant bleeding between the three study groups (2.7, 2.0, and 3.1%, p for trend = 0.99).

At discharge, as well as at 30 days of follow-up, patients in the three study groups were equally treated with anti-platelets (94, 97, and 97%, p for trend = 0.11), including P2Y12 antagonists (94, 97, and 96%, p for trend = 0.4), as well as lipid-lowering therapy (97, 99, and 99%, p for trend = 0.24). In contrast, patients with clinical PAD were less likely to receive angiotensin-converting enzyme (ACE) inhibitors and/or angiotensin receptor blockers (ARBs) (82, 90, and 94%, p for trend = 0.007).

### Mid-term outcome

**FIGURE 2:**
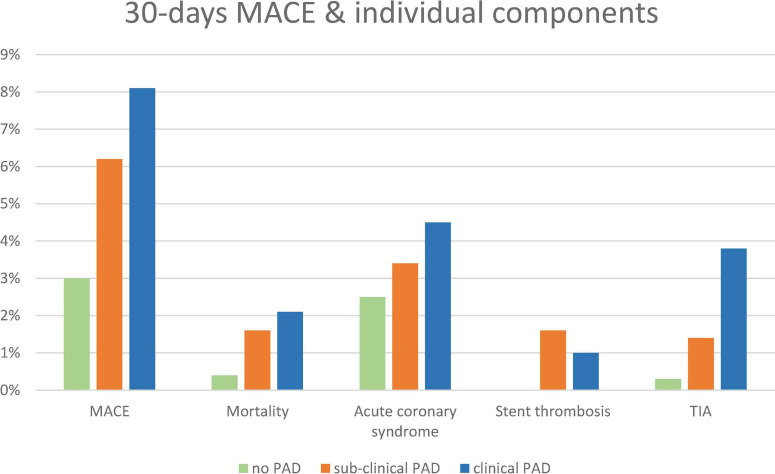
A 30-day MACE, individual components, and bleeding according to pre-specified groups: no-PAD (green), subclinical PAD (orange), and clinical PAD (blue). MACE, major adverse cardiovascular event.

At 30 days of follow-up, there was a stepwise increase in the pre-specified 30-day MACE rate, such that patients with no evidence of PAD had the lowest rate, followed by subclinical PAD and clinical PAD (3.5, 6.8, and 8.1%, p for trend = 0.006) (Figure 2). Accordingly, there was a trend toward an increase in the incidence of 30-day re-hospitalizations (14.7, 18.8, and 29.4%, p for trend = 0.082). The gradual increase in the pre-specified 30-day MACE was derived from an increase in the 30-day cardiovascular mortality rate from 0.4% in patients without PAD to 1.6 and 2.1% in patients with symptomatic and subclinical PAD, respectively (p for trend = 0.043), as well as an increase in the rate of recurrent ACS (2.5, 3.4, and 4.5%, p for trend = 0.17), stent thrombosis (0, 1.6, and 1.0%, p for trend = 0.013), and stroke and/or TIA (0.3, 1.4, and 3.8%, p for trend < 0.001). Although there was a trend toward an increase in minor bleeding (0.3, 0.8, and 1.9%, p for trend = 0.035), the incidence of major and/or medically relevant bleeding was equally low (0.1, 0.8, and 0%, p for trend = 0.8).

### Long-term mortality

**FIGURE 3:**
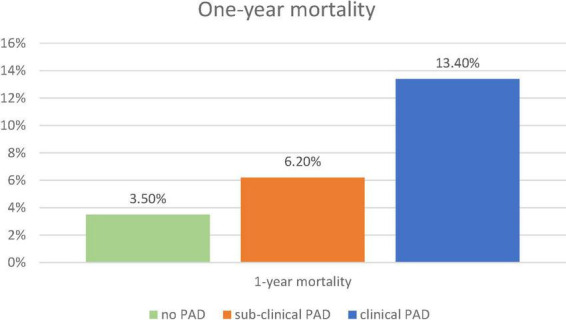
One-year mortality. The figure demonstrates the frequency of 1-year mortality according to the pre-specified groups: no-PAD (green), subclinical PAD (orange), and clinical PAD (blue). The chi-square test for trend was used. PAD, peripheral arterial disease.

During a 17-month post-discharge follow-up, 56 (5.3%) deaths occurred. There was a significant increase in 1-year mortality from 3.4% in patients without PAD, through 6.8% in patients with subclinical PAD to 15.2% in patients with clinical PAD (p for trend = 0.001, Figure 3). The mortality differences between patients with subclinical PAD and those without PAD did not reach a statistical significance (*p* < 0.09). Accordingly, the univariable analysis showed PAD (clinical or subclinical) to be associated with more than three times increase in the risk of mortality (hazard ratio [HR] 3.28, 95%CI 1.92–5.60, *p* < 0.001).

**FIGURE 4:**
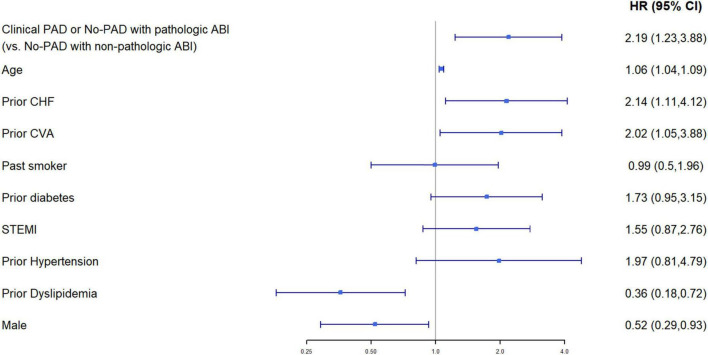
Multivariable Cox proportional hazard model for 1-year mortality (95%CI). Baseline characteristics (*p* < 0.05 for comparison between the groups of clinical PAD or no PAD with pathological ABI, vs. no PAD with non-pathological ABI) were chosen as covariates. Model’s data contain only patients with non-missing values in these variables, *N* = 1,035 patients.

**FIGURE 5:**
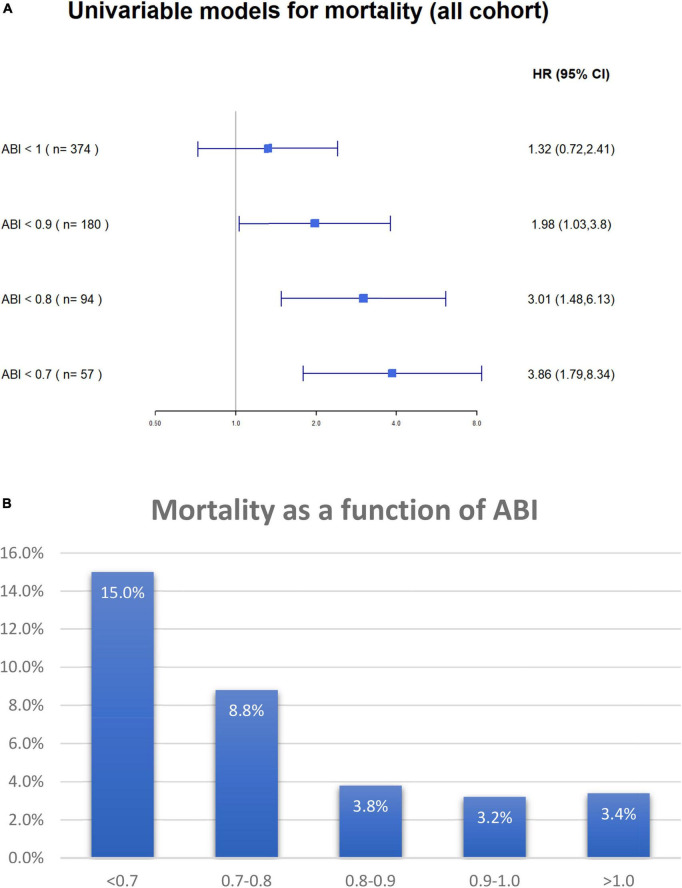
**(A)** Univariable Cox proportional hazard models for 17-months (maximal follow-up time) all-cause mortality according to ABI value. The figure demonstrates the difference in mortality according to ankle-brachial index cutoff points. N in each model (shown in parenthesis) regards to the number of patients with ABI less than 1, 0.9, 0.8, and 0.7 accordingly. ABI, ankle-brachial index. **(B)** Overall mortality according to ABI groups. ABI, ankle-brachial index.

Multivariable cox regression analysis, adjusted for age, gender, congestive heart failure, prior ischemic vascular event, smoking status, diabetes mellitus, hypertension, dyslipidemia, and ST-elevation myocardial infarction as the diagnosis of the index event, found an independent more than two-fold increase in the risk of mortality among patients with either clinical or subclinical PADs (HR 2.19, 95%CI 1.23–3.88, *p* < 0.001) (Figure 4). Both heart failure and prior stroke were associated with a similar 2-fold increase in the risk of mortality (HR 2.14 and 2.02, respectively). Both male gender and dyslipidemia were associated with a decreased risk of mortality (HR 0.52 and HR 0.36, respectively). Other factors did not reach statistical significance. As shown in Figure 5A, when patients were stratified using decreasing ABI values (from 1 to 0.7), lowering the ABI level used as a cutoff point was associated with a greater increase in long-term mortality among those patients who were designated as having abnormal ABI. In a univariable analysis, while ABI < 1 as compared to > 1 was associated with a non-significant (*p* = 0.4) 30% increase in mortality, ABI < 0.9 was associated with two times higher mortality (*p* = 0.05), ABI < 0.8 with three times higher mortality (*p* = 0.003), and ABI < 0.7 with almost four times higher mortality (*p* < 0.001).

When only patients without clinical symptoms of PAD were stratified based on their ABI values to those with ABI > 1, 1–0.9, 0.8–0.7, and < 0.7, there was a clear relationship between ABI stratum and long-term mortality (3.4, 3.2, 3.8, 8.8, and 15.0%, respectively, p for trend = 0.002) (Figure 5B).

## Discussion

The current study, conducted on a relatively large cohort of consecutive patients with ACS, has managed to show a stepwise increase in adverse events and mortality among patients with clinical and subclinical PADs, compared to patients without PAD.

The presence of PAD among patients with CAD is frequent with a prevalence of 22–42% (23–25). Among patients with stable CAD, those patients with PAD have higher cardiovascular adverse events (1, 13) and higher mortality (20). Moreover, mortality is inversely related to the severity of PAD (24).

In contrast to patients with stable CAD, the relationship between patients with PAD and ACS is less robust, and even less information excites regarding the significance of subclinical PAD in ACS. Clinical PAD is associated with an increased risk of mortality among patients with ACS during their index hospitalization (26) and long-term follow-up (27). In a retrospective analysis of the PAMI trials (28), which included 3,700 patients with ST-elevation myocardial infarction undergoing primary coronary reperfusion, 11% had clinical evidence of extra-coronary atherosclerotic vascular disease. Clinical PAD was associated with significantly higher in-hospital and 1-year MACE and was an independent predictor of in-hospital (odds ratio [OR] = 1.86, 95%CI 1.1–3.26) and 1-year all-cause mortality (OR = 1.7, 95%CI 1.1–2.75).

In these ACS studies (18–20), PAD was retrospectively defined according to symptoms or previous vascular interventions, and less attention was given to pathological ABI and subclinical PAD.

The impact of pathological ABI was evaluated in a large group of patients with either ACS or acute cerebrovascular events (29). Pathological ABI was associated with higher rates of non-fatal myocardial infarction and all-cause mortality. The predictive value of ABI was mainly accounted for by patients hospitalized with ACS. Although as many as 10% of the patients with ACS had clinical evidence of symptomatic PAD (intermittent claudication), the impact of pathological ABI was studied regardless of whether the patients had symptomatic PAD. Thus, the impact of ABI in patients with asymptomatic ACS was not disclosed.

Morillas et al. (2) evaluated 1,054 patients hospitalized with ACS, of whom 150 patients were diagnosed with clinical PAD and 298 patients were diagnosed with subclinical PAD according to pathological ABI, and found a stepwise increased risk of mortality among patients with subclinical PAD and clinical PAD. Both clinical (HR 4.38, 95%CI 1.96 to 9.82, *p* < 0.001) and subclinical PADs (HR 2.35, 95%CI 1.05 to 5.23, *p* < 0.05) were associated with higher 1-year mortality. Yet, a relatively low number of documented events were not sufficient to allow for an adequate analysis to adjust for the numerous differences in baseline characteristics between patients with and without PAD.

Our findings further support these observations and strengthen them by showing the independent impact of PAD whether symptomatic or subclinical (abnormal ABI) on outcomes after adjustment for multiple cardiovascular risk factors.

The observation that there is a stepwise increased risk for mortality among patients with subclinical and clinical PADs emphasizes the notion that PAD should not be regarded as a yes or no parameter, but rather as a continuous and ongoing parameter that adversely affects patients’ outcomes. The gradual increase in overall mortality with lower ABI values, shown in Figure 5B, further supports this observation.

In this study, patients with PAD and subclinical PAD had a significantly higher incidence of multivessel CAD and were more likely to have had diabetes mellitus. Accordingly, they were two times more likely to undergo CABG (10 vs. 5%). Thus, altogether, the incidence of coronary re-perfusion was only mildly reduced in patients with PAD as compared to patients without PAD. Moreover, both 1-year mortality that was the primary end point and 30 days MACE were demonstrated to be significantly higher in patients with PAD after adjustment in baseline characteristics, coronary findings, and interventions.

Several studies have evaluated the mechanism by which PAD adversely affects patients with heart disease (30, 31) including a higher prevalence of cardiovascular risk factors among patients with PAD who are poorly controlled, association with more severe and diffuse CAD, greater systemic inflammatory burden, and multiple arterial plaques.

More recently, it has been suggested that PAD is associated with greater inflammatory and pro-thrombotic responses, signifying more “malignant” atherosclerosis that might contribute to a worse prognosis in patients with both symptomatic and subclinical PAD. Among patients with CAD, PAD was associated with impaired endothelial function (32, 33), heightened inflammation (34), and a higher propensity toward thrombosis (35), as well as high on-treatment platelet reactivity (9).

Probably no single mechanism can explain the poor outcome of patients with PAD and subclinical PAD, but rather a combination of several factors that have an additive effect.

This study also found that both male gender and prior dyslipidemia were associated with a better outcome. Prior literature has shown that age, prior heart failure, prior cerebrovascular attack (CVA), and female gender were independently associated with a worse prognosis. In addition, those with prior dyslipidemia were significantly more likely to be treated with statins that might have a protective effect on clinical outcomes among those patients.

### Limitations

Although this is a prospective study, it is a non-randomized, unblinded observational study. In addition, it is subject to limitations that are inherent in the study design. However, this study represents a large unrepresentative cohort in 22 hospitals in Israel. Data for this study can be generalized to apply to most patients hospitalized with ACS. In addition, the designation of patients with PAD was according to a pre-specified set of criteria; however, we were not able to stratify the patients based on the specific event which entitled to patients with PAD (clinical claudication, demonstration of significant peripheral disease, and/or peripheral revascularization). Finally, we suggested that patients with both clinical and subclinical PADs might have more “malignant” atherosclerosis, from both thrombotic and inflammatory prospective. The study was clinical and, as such, did not include the determination of biochemical markers of thrombogenicity and inflammation.

## Conclusion

Subclinical and clinical PADs are associated with poor outcomes among patients with ACS, suggesting that routine ABI tests to enhance risk stratification might carry important prognostic significance in these patients regardless of PAD symptoms.

## Clinical perspectives

The findings of this study suggest that subclinical and clinical PADs are associated with poor outcomes among patients with ACS; thus, routine ABI tests to enhance risk stratification might carry important prognostic significance in these patients regardless of PAD symptoms.

## Data availability statement

The raw data supporting the conclusions of this article will be made available by the authors, without undue reservation based on a reasonable request from the corresponding author.

## Ethics statement

The studies involving human participants were reviewed and approved by the regional Ethical Review Board at each site approved the trial protocol, and the trial was conducted according to the principles of the Declaration of Helsinki. Institutional review board approval was obtained from all the participating centers and all patients provided signed written informed consent to participate in the study.

## Author contributions

AB substantial contributions to the conception and design of the work, analysis and interpretation of data for the work, drafting the work, provide approval for publication of the content, and agree to be accountable for all aspects of the work in ensuring that questions related to the accuracy or integrity of any part of the work are appropriately investigated and resolved. ZI, SF, SA, OB, AE, MG, and RB substantial contributions to the acquisition, revising the work critically for important intellectual content, provide approval for publication of the content, and agree to be accountable for all aspects of the work in ensuring that questions related to the accuracy or integrity of any part of the work are appropriately investigated and resolved. SM substantial contributions to the conception and design of the work, acquisition, analysis and interpretation of data for the work, revising the work critically for important intellectual content, provide approval for publication of the content, and agree to be accountable for all aspects of the work in ensuring that questions related to the accuracy or integrity of any part of the work are appropriately investigated and resolved. All authors contributed to the article and approved the submitted version.

## Abbreviations

ABI: ankle-brachial index
ACS: acute coronary syndrome
ACSIS: Acute Coronary Syndrome Israeli Survey
CABG: coronary artery bypass graft
CAD: coronary artery disease
MACE: major adverse cardiovascular event
PAD: peripheral arterial disease
PCI: percutaneous coronary intervention
TIA: transient ischemic attack.
