# Supplementary figures and images for: Peripheral artery disease, abnormal ankle-brachial index, and prognosis in patients with acute coronary syndrome
Source: Front Cardiovasc Med. 2022 Sep 6;9:902615. doi: 10.3389/fcvm.2022.902615 (PMC9485724; doi:10.3389/fcvm.2022.902615)

eFigure 1 – flow chart


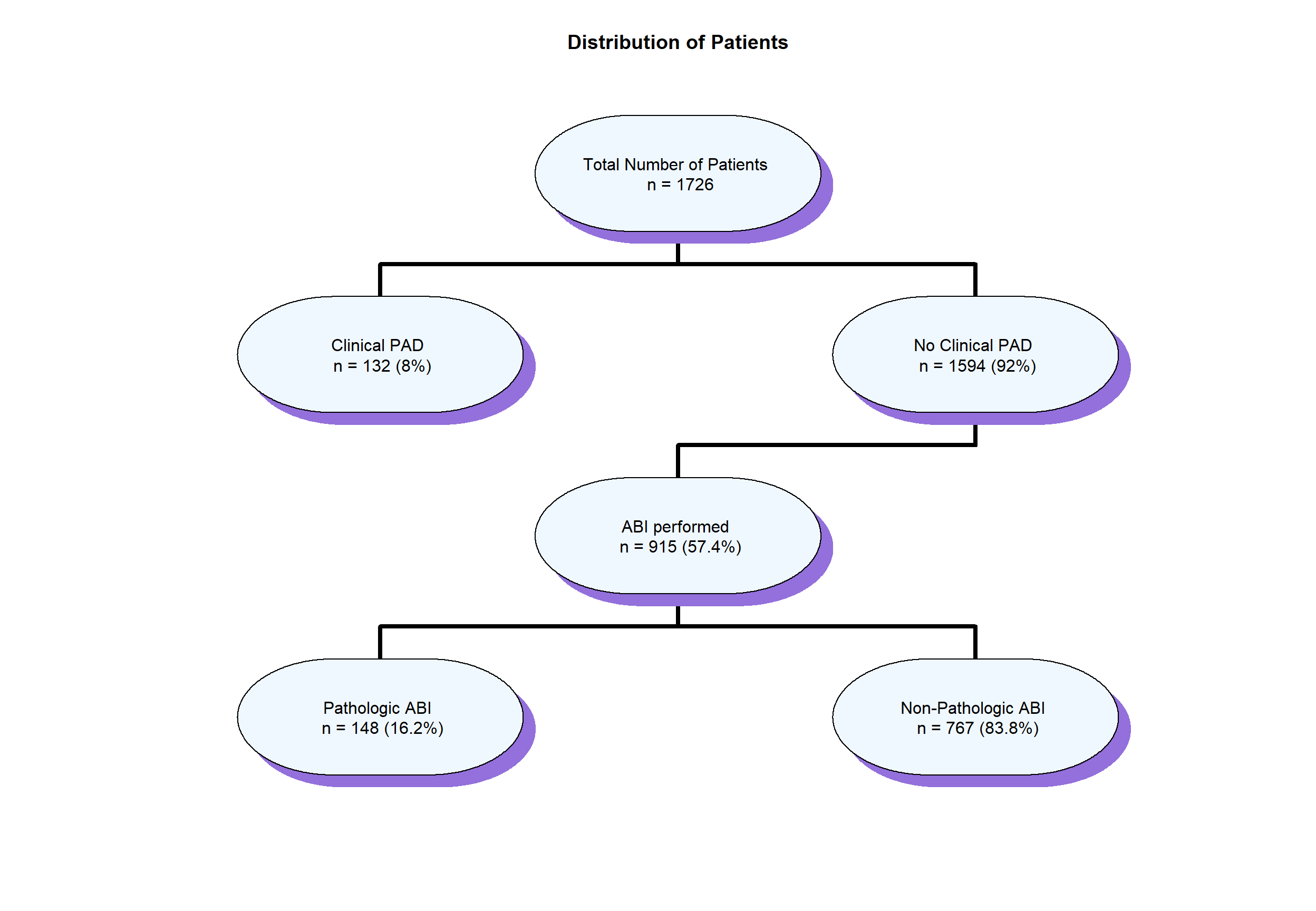


eFigure 2 –


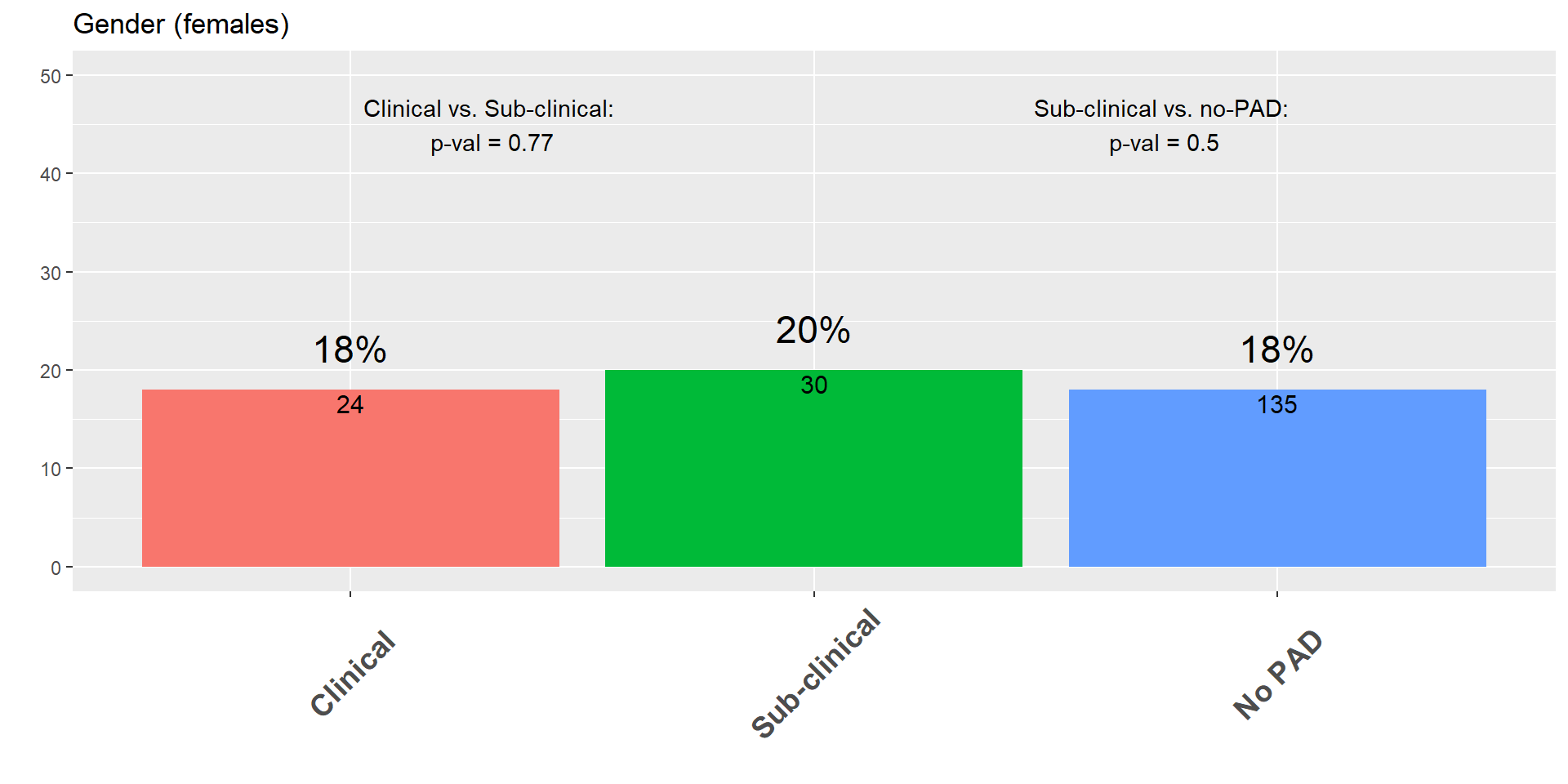

Supplement: Supplementary file 1 [file Data_Sheet_1.docx]
